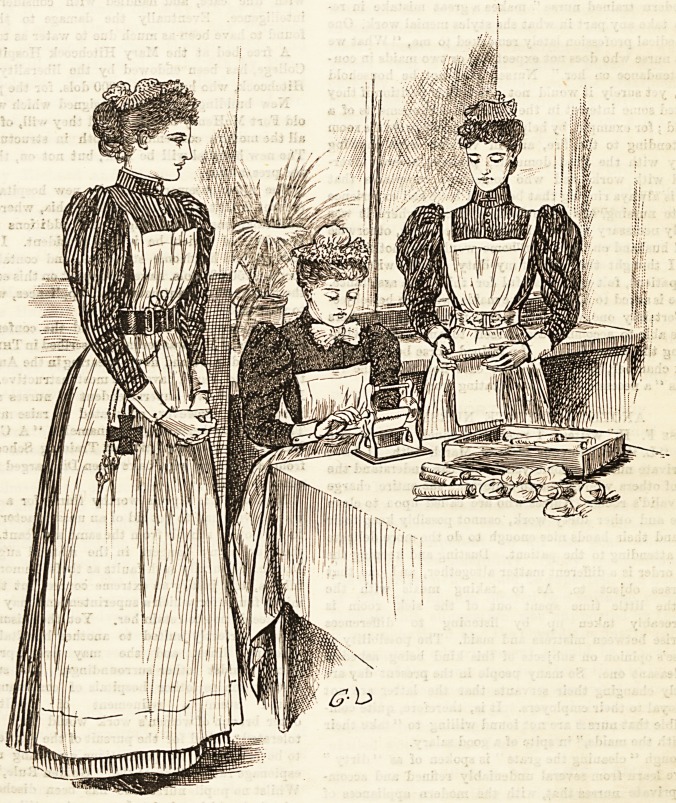# The Hospital Nursing Supplement

**Published:** 1895-04-13

**Authors:** 


### "The Hospital" Nursing Mirror.

Being the Extra Nursing Supplement of "The Hospital" Newspaper.

[Contributions for this Supplement should be addressed to the Editor, The Hospital, 428, Strand, London, W.C., and should have the word "Nursing" plainly written in left-hand top corner of the envelope.]

### News from the Nursing World.

### WITH THE SICK CHILDREN.

H.R.H. the Duchess of Fife enjoys no inconsiderable share of the affectionate popularity which surrounds her mother, "Our Princess." In the new wards at the Hospital for Sick Children, Great Ormond Street, opened by their Royal Highnesses the Prince and Princess of Wales two years ago, the Duke and Duchess of Fife last week spent some time talking with the little patients. The kindly gifts of toys, which they presented themselves to the children, brought bright smiles to many delicate faces, and it will be long ere the friendly visit of the President of the hospital and his Royal wife dies out of the memories of the small creatures whose mental precocity is in startling contrast to their physical condition. The important moment when they were noticed by "the Queen's very own grand-daughter" is already established as the date from which past and future events are reckoned.

### WESTMINSTER HOSPITAL BAZAAR.

The Nurses' Bazaar, at Westminster Hospital, was opened by Lady Knutsford on April 3rd, after a short speech had been made by Lord Knutsford, vice-president of the institution. Sir Rutherford Alcock and Dr. Allchin also spoke, and the nurses were warmly congratulated on the number of pretty and useful articles made by them. This sale of work doubtless added substantially to the funds of this well-known hospital.

### HELP SORELY NEEDED.

The Workhouse Infirmary Nursing Association is sorely in need of funds, and we learn with regret that their work is being seriously crippled by lack of means. When Guardians, convinced that trained nurses are essential, apply to the W.I.N.A. to supply them, it is with infinite regret that their applications are refused. Seventeen nurses were asked for in a fortnight. Since the beginning of the year two-thirds of the requests have had to be denied solely because the association has not sufficient nurses nor sufficient money for the training of fresh ones. Yet the modest sum of £20 represents the average cost of each nurse's training. Surely a subscription would be a practical accompaniment to the sympathy of many whose kindly interest has been awakened by the urgent needs of the sick poor in workhouse infirmaries !

### DISTRICT NURSING AT HAGGERSTON.

The valuable work of the Haggerston and Hoxton District Nursing Association is familiar to those who know the poor neighbourhoods in which it is carried on. The association, affiliated with the Queen Victoria's Jubilee Institute, held its fifth annual meeting, by the kind permission of Lord Meath, at 83, Lancaster Gate. The increase in local subscriptions is rightly looked upon as a satisfactory sign of progress, and the number of cases nursed, to which no less than 17,379 visits were paid during the year, proves the universal appreciation accorded to the trained and experienced nursing staff. The meeting was addressed by the Rev. C. J. Ridgeway, Mr. Herbert Robertson, and others, Dr. Hawkes cordially advocating the claims which the association had on the public. Patients could now, in many cases, be successfully treated in their own homes, and, from a medical point of view, he was glad to show that they were helped to live in a more healthy and cleanly manner.

### THE LAST THING IN BADGES.

The gauze veil, alternately praised and abused by the critics, has been promoted to the rank of a badge in Dorsetshire. Whether any definite period of training is demanded of the nurses who are there adorned with this novel form of certificate does not transpire; but, at any rate, the homely village "helpers" are denied the use of the distinctive veil! An extremely limited course of instruction enables the woman to adopt the imposing title of "village nurse," but at the same time it permits of no indulgence in gossamer on the simple and becoming straw bonnet, with white strings. On the whole, the plain cottager has the best of it, and on windy wet days her superior in training must become her inferior in point of professional neatness

### BAZAAR AT DERBY.

The bazaar in aid of the Derby Infirmary will be opened on April 16th by H.R.H. the Duchess of Teck, accompanied by the Duke. There seems every prospect of the infirmary being substantially benefited by the entertainment which has been organised by friends of the institution.

### PROMISCUOUS CHARITIES.

Against the use of collecting-boxes which irresponsible youngsters thrust suddenly under the noses of startled pedestrians, warnings have been frequently uttered. Of the ultimate destination of the unwillingly contributed coins many persons are exceedingly doubtful. A fresh nuisance in the collecting line has developed at Bristol, where an old patient from the General Hospital supplied himself with a box, a book, and the other paraphernalia associated with public almsgiving. The hospital itself, a nurse, and his own health alternately furnished an object of appeal to a kindly public. The General Hospital authorities objected to the ingenious collector passing himself off as their under secretary, and the magistrate, disregarding his expressions of regret, committed him for trial at the Quarter Sessions, as other charges of obtaining money under false pretences have been made against Mr. G. A. Godby.

### "A YOUNG PERSON" AS A NURSE.

Not a single application had been received in answer to their advertisement for an assistant nurse, was the report of the chairman of the Loughborough Board of Guardians last week. The candidates who haunt some institutions were apparently not attracted by this vacancy, and the Guardians rested content with the fact, without searching for the cause of their failure. An informal application from "a young person" was then received by the board, who proceeded, after interviewing the doctor and matron, to appoint this candidate nurse of the women's ward. She had acted hitherto only as industrial trainer, and the matron had another "young person" to recommend as her successor. It is quite possible that the Local Government Board may not sanction as easily as the Guardians have done, the substitution of "industrial" experience for training in sick nursing. At any rate, for the sake of the helpless poor, it is to be hoped that such arrangements will be inquired into.

### KILMARNOCK.

There was a large attendance at the annual meeting of the Kilmarnock Nursing Association last month, and the report of the work accomplished by the three district nurses was eminently satisfactory. They had paid 10,818 visits to 356 patients, and their services were increasingly sought after. Votes of thanks were accorded to the Working Committee, the Lady President, Mrs. Ferrier Hamilton, and to the Honorary Secretary, Mrs. M'Alister.

### WHO ATTENDS HEALTH LECTURES?

In the country it is certainly the leisured people who chiefly patronise health lectures, the great proportion of seats being appropriated by the ladies of the neighbourhood. If the free lectures on nursing and kindred subjects are to benefit the poor, surely more trouble might be taken to secure the presence of those who cannot pay for such instruction. The shyness of the rural population keeps them from coming forward to be taught, although their presence once secured they make excellent pupils. Even in towns there seems a difficulty in getting together the right sort of audience, and it is evidently desirable that lady lecturers should attract more of the rising generation, the wives and mothers of the future, instead of resting content with the middle-aged women who come in to see if the speaker "has anything new" to tell them; or the ladies who get "free education" in preference to paying for classes.

### SYMPATHETIC OR ANTAGONISTIC?

It may well be questioned how far women guardians realise their responsibilities with regard to the female officers of workhouses? In but few cases, for instance do they estimate the enormous assistance they render to infirmary matrons and to the patients if they are equally considerate of both. With the ignorance of inexperience, guardians are apt, with the best intentions in the world, to fancy themselves to be protectors of the rights of patients, without always troubling to discover in what the said "rights" consist. By friendly combination with the matron and charge nurses many improvements might be inaugurated by women guardians comprehending the difficult duties which these officers have to cope with single-handed. A little reflection would enable any intelligent woman to realise a nurse's true position and a matron's grave responsibilities. Only when this is done can the full advantage of having "women on the Board" be secured in a poor law infirmary. It is peculiarly desirable at present to urge these ladies to realise the nature of their duties and responsibilities, for 45 out of 83 recently elected in London are reported to be new to the work. Wales has 88 women guardians, and the total number in England (including the metropolis) and Wales now stands at 830.

### A TRAINED NURSE.

The Newark Guardians want a trained nurse for their union, which contains an average of 70 inmates, including 19 children. Of the latter this trained nurse is to be also the caretaker, which post is explained as looking after the children when not in school, she will also be privileged to entirely manage their clothing. Her qualifications for trained nursing will presumably enable her to fill up her spare time by attendance on the sick or infirm. For all this work a salary of £20 per annum is offered, with washing, coals, light, and furnished apartments, and *such rations as may be mutually agreed upon.* We wonder if these rations can be made equivalent to a "full diet."

### HOLLAND.

It is proposed, this year, to hold a somewhat novel exhibition at the Hague, from July 8th to the 17th. It will consist solely of various kinds of plants and herbs which are used medicinally, and several doctors are on the committee of management. The society, which assists convalescent children after they have been discharged from hospital, issues a pressing appeal for funds which are urgently needed as the sphere of the society's work continues to widen. Children who are too small or weak to come regularly to the headquarters in Prinsenstraat are visited at home. Quantities of eggs, milk, soup, bread, oatmeal, &c., are dispensed to the convalescents, and the expenses continue to grow without any corresponding increase of regular income. Happily, an unexpected windfall enabled the society to end the year 1894 free from debt.

### SHORT ITEMS.

The Hewell and District Nursing Association continues to do good work, three trained nurses being employed.—The Bath Guardians have agreed to wait for nurses to be supplied to them by the Workhouse Infirmary Nursing Association, for the staff of which Miss Robinson has been appointed superintendent nurse.—The Marchioness of Londonderry presided over a meeting of the Stockton and Thornaby Nursing Association last week.—Miss Stewart MacDonald, of the Liverpool Northern Hospital, is succeeding Miss Hassall as parish nurse at Crediton.—A petition from Miss Ada Richardson, regarding a trained nurse for Alton, was laid before the Alton District Council, accompanied by a memorial signed by the medical men and ministers of the district; in spite of this influential support a majority voted "that the matter remain in abeyance."—The Hucknall and District Nursing Association held its first annual meeting last week and laid a satisfactory report before the subscribers, and the trained nurse and probationer employed by the association have paid 1,663 visits to ninety-four patients.

### Elementary Anatomy and Surgery for Nurses.

By W. McAdam Eccles, M.B., M.S., F.R.C.S., Lecturer to Nurses, West London Hospital, &c.

### XII.—THE DIGESTIVE SYSTEM.

In order that the food which is eaten may become of use in nourishing the body and renovating its tissue it is necessary for it to be variously modified. These changes are brought about in the alimentary tract, which must be followed from its commencement to its termination, and the various secreting and other glands associated with it spoken of.

Fig. 19.—Upper Surface of Tongue.

The tract begins with the mouth. This cavity is guarded in front by the lips; within these is the double row of teeth. It is well to remember their names and order as given in Lecture III. On the floor of the mouth is the muscular organ called the tongue. It is very freely movable, and will be seen to be covered with papillæ, which are of three varieties, some long and slender, and at the same time the most numerous, the filiform; others conical or shaped like a mushroom, much fewer in number, the fungi-

Fig. 20.—Section of Face and Throat.

1, Pharynx; 2, gullet; 3, vertebræ of neck; 4, nasal passages; 5, soft palate; 6, tongue; 7, epiglottis; 8, windpipe.

form; and lastly, at the back are some seven or nine arranged in two rows like the letter V, these are the circumvallate papillæ, each being surrounded by a kind of wall with a depression or moat within (see Fig. 19).

The mouth has on either side the inner surface of the cheeks, and above will be observed the hard palate, to the hinder part of which is attached the soft palate hanging somewhat like a curtain, and partly closing the aperture between the mouth and the pharynx. The soft palate has two folds on either side, termed the anterior and posterior pillars of the fauces. Between these the tonsil lies, and hanging in the middle line is the uvula.

The pharynx is the conical-shaped cavity behind the mouth. It has seven openings into it, namely, the mouth, the larynx or commencement of the windpipe, the œsophagus or gullet, the two posterior nares or nostrils, and the two Eustachian tubes which pass from the naso-pharynx to the ears.

The œsophagus is that part of the digestive tract which lies between the pharynx and stomach. It is about nine inches long. In its course it traverses the thorax lying behind the heart, and pierces the diaphragm to enter the stomach (see Fig. 21).

Fig. 21.—The Alimentary Tract.

cs, Fundus of stomach; s, greater curvature of stomach; œ, œsophagus; sp, spleen; p py, pyloric end and pyplorus of stomach; d, duodenum; i, small intestine; l, liver; gb, gall bladder; va, vermiform appendix; c, cæcum; ac, ascending colon; tc, transverse colon; dc, descending colon; ic, sigmoid flexure; r, rectum; a, anus.

The stomach is a large hollow muscular organ situated in the upper part of the abdomen just below the diaphragm. Most of the parts of the tract under consideration which lie in the abdominal cavity are covered with a smooth shiny membrane named the peritoneum, which serves to allow them to glide easily on each other without friction. The inner surface of the abdominal walls is also lined by the same membrane. Beneath this serous coat is a layer of muscular fibres which are unstriped in character and arranged in different directions, but chiefly so as to encircle the organ, and within this muscular coat is the mucous membrane which secretes digestive fluid, called in the case of the stomach the gastric juice. The enlarged left end of the stomach is called the fundus or cardiac extremity (see Fig. 21); the narrower right end is the pylorus, at which the small intestine begins. The upper border of the stomach is short and concave—the lesser curvature; the lower long and convex—the greater curvature, from which hangs a fold of peritoneum, the great omentum lying like an apron in front of the small intestine. The stomach comes in contact in front with the left lower ribs, the left lobe of the liver, and part of the anterior wall of the abdomen.

### Invalids at Netley.

Little do those dwellers in peaceful English homes dream of the fight that many soldiers have to endure with the disease of tropical stations simply to secure ease and peace for us. A draft of healthy young men sent out, say to India, have first to pass a medical examination. They are, as a rule, all supposed to be twenty years of age, and, alas! many will never see their native land again, many more will return mere broken-down wrecks of their former selves.

At Netley, when a batch of invalids is expected, all empty beds are carefully noted; each sister in charge of a division having eighty beds under her care. In a civil hospital, a sister would be probably surprised to learn that twenty or thirty admissions would take place in one evening. It is a sad, if interesting sight to watch the sick brought in. So many "cot cases" are reported, and so many convalescents. The "cot cases" are those admitted to the troopship in a helpless condition; some of these improve by the voyage home, and are in better condition than the convalescents. Many of the latter, without hospital comforts on the troopship, and getting only "ship's food," go rapidly down hill, and when they reach Netley are only just able to crawl to their last resting-place. As many ambulances as are asked for meet the invalids at the railway station, about a quarter of a mile from the hospital; those who are considered able have to walk. As soon as the patients arrive they are told off to their respective wards, and they settle down quickly with very little confusion. Each man has his full hospital kit given to him. This consists of flannel and cotton shirts, trousers, jacket, greatcoat, cap, necktie, pocket-handkerchief, sheets, pillow slip, towel, brush and comb, crockery, knife, fork, and spoon. The sister sees to all bad cases, making their beds, generally helped by convalescent patients, as the orderlies are busy taking the regimental kits into stores and settling numerous little matters. These include baths for convalescents; foot warmers for ague cases or others needing them. Then the orderly medical officer comes round and orders tea and bread and butter and extractum carnis, and beef tea for convalescents. He finds out from the sister what milk is required for bad cases, stimulants, and medicines and treatment where necessary. Then quiet reigns, and the sister goes to each case and finds out as much as she can about them and gives the prescribed nourishment and treatment. Perhaps she finds a supposed convalescent unable to take bread and butter, then she either gets him bread and milk, or milk only. She is more than likely to find a bad heart and dropsy case that she had left tucked safely up in bed quietly seated on a form at the table with his white quilt wrapped round him playing cards. Perhaps some dying lad sobs out a request that his mother may be sent for. Sad are the tales of sufferings on the way home. One lad in a consumption had a hammock swung in a doorway so that he might get more air, "but somehow men were always passing, and always managed to bump against it" till ribs and back ached. Another case of debility and emaciation after typhoid fever had a "wind bed" in which "the wind never stayed." Numerous pets are brought home by the men—parrots, monkeys, &c.—and there is a special place in the bath-house at Netley where they are taken care of. There is also a covered swimming bath for convalescents, and nice grounds and a pier where all who are allowed out can get exercise. The wards for the most part average about ten beds, and there is an orderly to each ward. Unfortunately the wards are built on the wrong side of the hospital, so the long corridor with glass front a quarter of a mile in length is a sunny place, but the wards seem dull and grey in appearance. However, good work is done there; broken-down constitutions are patched up, health is restored, or dying beds eased by tender care. Invalids from all regiments except the artillery come to the Royal Victoria Hospital, Netley. The knowledge of that being his destination raises hope in the breast of many a returning soldier.

### District Nursing at Worthing.

The Mayor of Worthing presided over the annual meeting of the District Nursing Association, which was recently held in the Steyne Assembly Rooms. The association (affiliated with the Queen's Jubilee Institute) is greatly appreciated by doctors and patients, and the year's record of work is an excellent one. Miss Hughes, of the Central Training Home, Bloomsbury, gave an excellent address, in which she explained her strong personal interest in this association, as all the nurses employed by it had been sent by her. Therefore, the success of their work and its steady growth were indeed matters of personal congratulation. Miss Hughes went on to explain the place which skilled nurses filled, and deprecated the ignorance which caused some people to imagine their duties were identical with those of charwomen. It would help people if they started with the conviction that disease was not a necessity, and that it was not a thing to be made the best of, but that it was a thing they ought to prevent. They had to go into the homes of the poor and try to make the inmates realise their mistakes, to tell them that a great deal of disease was caused by want of ventilation, overcrowding, and want of proper food. They wanted their nurses not only to go about as working machines, trained by good drill to do their work, but as "health missionaries," as Miss Nightingale called them. District nursing was one of the most civilising agents of the present time, Miss Nightingale said. It was not always very interesting, especially going day after day to a patient and finding nothing different. Nurses needed encouragement. Miss Hughes spoke of the importance of training. However anxious a nurse might be, she wanted training to keep up to the ideal. She had to take into the people's homes knowledge and a better standard. In order to do that special training was required. Miss Hughes's address was received with close attention, and was such an excellent sketch of what district nursing is and should be that her hearers passed an enthusiastic vote of thanks for her visit. Dr. Hinds, the Rev. F. C. Cass (president of the association), the Mayor, Mr. Merriman, and Mr. King were also speakers at this meeting, which should result in a large increase in the subscription list.

### Where to Go.

International Exhibition of Hygiene in the Palais des Arts Libéraux, Paris, May 15th to September 15th.

Mildmay Mission.—Aunt Esther's Cot Fund. A sale of work in aid of this fund will be held at 15, Craven Hill Gardens, Hyde Park, W., on Wednesday, May 29th, from 12 to 6 p.m.

Trained Nurses' Club, 12, Buckingham Street, Strand.—At 7.45 on Friday, April 26th, Dr. G. E. Herman will lecture on "The Consequences of Mismanaged Labour." Tickets can be procured by non-members at sixpence each.

St. Martin's Town Hall.—Annual meeting of the Zenana Bible and Medical Mission at 3 p.m. on April 23rd. Tickets can be obtained of the secretary, 2, Adelphi Terrace, W.C.

Kensington Town Hall.—On May 1st, from 3 to 6.30, and from 8 to 10.30, "A May-Day Masque" and Café Chantant will take place in aid of the funds of the Hospital and Home for Incurable Children, 2, Maida Vale. Tickets for afternoon, including tea, 2s. 6d. Admission in evening, one shilling.

### A Great Movement: The Nurses' Co-operation.

III.—RESULTS: HOW WOMEN CAN CO-OPERATE.

The work of the Co-operation has been steadily increasing through the years. In 1891 there were 1,127 cases attended, in the following year 1,732, while in 1893 the number rose to 2,705. Naturally the nursing institutions, seeing the success of the new-comer, became restless. The Co-operation was injuring them, but they could not attempt to compete with it, unless they changed their principles entirely. It was a new form of rivalry. They were not undersold, the sick world was not supplied with an inferior quality of nurse at a cheap rate, which might have accounted for a sudden and temporary success. The fees were as high as ever, the Co-operation nurses, to say the least, as good as the institution ones, and yet without such claims on public support, the Co-operation flourished. As a matter of fact, many people who had had nurses had paid the institution fees reluctantly, not because they grudged them to the nurse, but because the nurse received so small a portion of them. Opportunity being given, most people will get rid of middlemen's profits, and patients who were personally grateful to the nurses who had tended them in sickness resented the sweating system of the institutions, and were glad to facilitate the nurses getting the money they earned. It is this desire on the part of the public to pay its debts where they are due that has been the bulwark of the Co-operation, and to compete with it at all the sweating institutions had to deal more generously—we ought to say less unjustly—with their nurses. Some raised their wages; others gave them a percentage of their earnings in addition to their meagre salary. The Nurses' Co-operation has undeniably been a friend to other nurses than those on its own staff, and this is not the least of its merits. Much thought, much generosity, and much unselfish labour were bestowed on it before it came before the world, and nothing would gratify its founders and promoters more than the knowledge that their efforts had benefited the whole class of "ministering women," and not only those on their own staff. In other towns co-operations sprang up and sought affiliation with the London one. It was not thought advisable to accede to these requests. There was nothing to be gained by it for either party. The object of the Co-operation was to give nurses as much as possible of their own earnings, and there was no prospect of the expenses of administration being diminished further by this affiliation; indeed, the reverse was likely to be the case. Therefore the applications were declined, while at the same time the committee of the Co-operation rejoiced to see its example so quickly followed.

Besides these genuine imitations of the Association in New Cavendish Street, there arose co-operations where there was nothing co-operative but the title, and nurses, too hastily joining these, have suffered disappointment and loss. Work has been promised by self-appointed officials, who had no power to secure it for their candidates. For it must be remembered that the approval and support of influential doctors in good practice was necessary to the success of the scheme. Nurses alone could not have created and sustained the Association without doctors to recommend it, and draw their helpers from its ranks. Where it is not backed by the chief doctors of the neighbourhood in which it is placed, a co-operation, whether founded in good faith or not, is predoomed to failure. It is essential also that the nurses should maintain a high standard of efficiency in whatever branch of work they profess. A co-operation must justify its claim to public confidence. Therefore it must not consist of any heterogeneous collection of women, skilled or unskilled, as chance may fall, and perhaps lacking in those moral qualities which are among the equipment of an acceptable nurse. In short, a nurses' co-operation means more than a mere conglomeration of nurses.

We may expect to hear from critics of these failures, perhaps from those who have been losers by them, that the co-operation of nurses is a failure. This is not so; but to secure the success which the original Co-operation has won several things are necessary. First, the support of influential doctors. Second, a sufficient number of nurses to join it. So many of the expenses are unvarying that, the larger the number the smaller the percentage that requires to be deducted to pay them. Rent is the same, so are rates, and the cost of gas and firing, whether the office is the bureau of ten or a hundred. The salaries of officials and their number may increase with the number of members, but not in anything like direct proportion. Speaking generally, a large business can be wrought at smaller expense than a small one. In the case, however, of such an organisation as the one we are speaking of, the number of members must be limited by the necessity for the executive committee having some personal knowledge of those they admit. Thirdly, nurses must cultivate sufficient knowledge of business to understand why they must give up some portion of their earnings for office expenses, and why they must hang together as men do, instead of underselling each other either for selfishness or so-called charity. "Ye have the Pyrrhic dance as yet; where is the Pyrrhic phalanx gone?" The Pyrrhic dance is a graceful amusement for idle women, but when they take to earning their bread, it is more important that they should learn the lesson of the Pyrrhic phalanx—that standing shoulder to shoulder which is the secret of men's success. Fourthly, they should have confidence in their officials, and should therefore choose those in whom they can reasonably have confidence. They must learn that misfortune is not a passport to authority. The man or woman who has not made a success of his or her personal career is not the one to put in a position of responsibility. The fatal tendency to appoint persons to positions of authority because of their needs rather than of their capacity is one of the most frequent causes of the failure of feminine undertakings.

But given these essentials, there is no reason why in other towns co-operations should not succeed. What success has attended the parent one may be inferred from the fact that the business done by it last year exceeded £30,000 in value. Several honorary workers have given place to paid ones, but the Co-operation can afford to pay, while from time to time the members have testified to their appreciation of those who have worked for them by handsome gifts. Some have devoted tithes of their earnings to the decoration of the sitting-room, and can afford to do so, for their earnings average about £85 a year. They are able also to take good long, refreshing holidays, and to make provision for sickness and old age, by belonging to the Royal National Pension Fund for Nurses, which all would be wise to join, and especially for sick pay.

### Appointments.

Cardiff Union.—Miss Charlotte Mary Williams has been appointed Superintendent at the Cardiff Union Hospital by the Workhouse Infirmary Nursing Association. Miss Williams was trained for three years at Brownlow Hill Infirmary, Liverpool, where she was also night superintendent, and she held the post of night superintendent at the Royal Infirmary, Edinburgh, for two years. Many good wishes for success in her new and responsible position will accompany Miss Williams to Cardiff.

### Addenbrooke's Hospital, Cambridge.

The New Nurses' Home.

We congratulate the governors of Addenbrooke's Hospital upon the decision arrived at, at the quarterly court held on the 25th ult., when it was determined to build a new Nurses' Home upon plans prepared by the architect, Mr. Fawcett. The new Home will, we understand, contain ample accommodation for thirty-six probationers, in addition to rooms for the night superintendent. On the ground floor there will be a recreation and general sitting-room for nurses; a quiet room or study where probationers can read under the most favourable conditions; a library well supplied with books; sitting-rooms for the night superintendent and the assistant matron; a box-room, cloak-room, two bath-rooms, and a certain number of bed-rooms. The first and second floors will be given up entirely to bed-rooms, each nurse being provided with a separate room to herself; bath-rcom three on each floor, lavatories, and the usual applisnc:s. We are glad to learn that the fault of many nurses' homes will be avoided, and that the bed-rooms will be of ample size, namely, not less than 9 feet by 12 feet. Although the Home is to be heated by hot water, it is satisfactory to know that each bed-room will contain a chimney for purposes of ventilation, although there will be no fireplace. One great feature will be the ample lavatory provision, the present accommodation being most defective in this respect. The existing Home will be devoted to the service of servants and wardmaids, the sick-room and its appurtenances being left as at present. We are glad to see, despite the carping criticisms of the mis-called economists, that the quarterly court decided *nem. con.* to vote the extra expenditure necessary to secure that adequate accommodation shall, at last, be provided at Addenbrooke's for the nursing staff. All who take an interest in this noble institution will rejoice that the new Home about to be erected is to be so planned as to make it one of the most complete, if not the most complete, building of the kind at present erected. If this object be successfully attained, Addenbrooke's Hospital will be placed in the forefront of modern institutions, with nurses' quarters worthy of the institution, and of any developments which nursing is likely to make in the present generation.

### Notes and Queries.

The contents of the Editor's Letter-box have now reached such unwieldy proportions that it has become necessary to establish a hard and fast rule regarding Answers to Correspondents. In future, all questions requiring replies will continue to be answered in this column without any fee. If an answer is required by letter, a fee of half-a-crown must be enclosed with the note containing the enquiry. We are always pleased to help our numerous correspondents to the fullest extent, and we can trust them to sympathise in the overwhelming amount of writing which makes the new rules a necessity. Every communication must be accompanied by the writer's name and address, otherwise it will receive no attention.

### Queries.

(115) *Massage.*—Will you give me some information as to training and certificate in massage?—*Tara.*

(116) *Midwifery.*—Information wanted respecting the gaining of certificate by a nurse with practical knowledge.—*Wood.*

(117) *Hospitals.*—Where can I get a list of nurse training schools?—*Beata.*

(118) *Hirsuto.*—Could you kindly inform me where I could obtain instruction in electrolysis, with a view to becoming a practitioner for the treatment of superfluous hair?—*Hospital Reader.*

### Answers.

(115) *Massage (Tara).*—See paragraph in The Hospital of April 6th. You had better write to the Hon. Secretary of the Society of Trained Masseuses, 18, Buckingham Street, Strand, London, sending a stamped envelope for reply.

(116) *Midwifery (Wood).*—You had better ask the Secretary of the Midwives' Institute for advice. If you write, enclose stamped envelope. Address Hon. Secretary, Midwives' Institute, 12, Buckingham Street, Strand, London.

(117) *Hosp.ta's (Beata).*—See "Burdett's Hospital Annual."

(118) *Hirsuto (Hospital Reader).*—The removal of hairs by electrolysis is a surgical proceeding which is best left to surgeons.

### Burdett's Official Nursing Directory.

An attempt is being made by those interested in the publication of a private list of nurses to dissuade nurses from entering their names in the Official Directory. Many interesting but incomplete and inaccurate details are given regarding Mr. Burdett, which, while obviously meant to prove his utter incapacity for the task of editing an Official Nursing Directory, will to most people appear good evidence of his special fitness for the work. It is stated that he was in his youth a medical student, studying at Guy's, one of the largest of our hospitals; that he has also been secretary of two hospitals; and that he now occupies a recognised position in the City of London connected with a public body through whom the largest financial business of the country is conducted. What more can be asked for in the conductor of such a publication as is here in question? A knowledge of medicine, an official knowledge of hospital management, a familiarity with business methods, a publicly recognised position—Mr. Burdett ought to thank his detractors for their description.

For, after all, knowledge, business capacity, and impartiality are the essentials for the enterprise, and it is childish to pretend that nurses and the public will not prefer a directory in the conduct of which these essentials are brought to bear to a directory issued by a mere party, which professedly will only contain the names of a section of the nursing profession.

While certain private individuals are striving to make it appear that nurses who are not registered by the British Nurses' Association are outside the pale and should no longer be recognised as nurses, Mr. Burdett is merely stepping in to furnish both the nurses and the public information which they want.

The Official Directory will be a directory pure and simple. The names of all the members of the British Nurses' Association may, and probably will, be found in its pages, but so will those of nurses who are not members of that body. To be in this Official Directory is a mere business matter. Other directories have failed because insertion in such lists meant belonging to a clique, while the great mass of nurses merely want to earn an honest living, and to do some good in their generation, and sections and divisions in their profession are to them anathema.

Burdett's Official Directory promises not to fail, because it is conducted on business principles and gives the information that is wanted, with no *arrière pensée.* We are informed that at a meeting of the Editorial Committee last week a surprisingly large number of nurses and institutions intimated their recognition of the importance and value of such a Directory, and announced their intention of doing their utmost to support the movement, and make it a success.

### Presentation.

The Executive Committee of the Workhouse Infirmary Nursing Association have given Miss Vause a handsome clock with an inscription: "Presented to Miss A. E. Vause, on the occasion of her marriage, by the Committee of the Workhouse Infirmary Nursing Association in recognition of ten years' loyal and devoted service." Miss Vause was trained by the association at Brownlow Hill Infirmary, Liverpool, in 1885, and subsequently at Kensington Infirmary in midwifery, taking the L.O.S. diploma. She worked as a member of the Association at St. Marylebone Infirmary and Stoke-on-Trent and Whitechapel Union Infirmaries. At the latter she held the post of night superintendent. Her last engagement was as superintendent of the Cardiff Union Infirmary, a post which she held for nearly four years, her admirable work of organisation there being fully recognised by the Guardians and medical officer.

### Dress and Uniforms.

By a Matron and Superintendent of Nurses.

### ST. THOMAS'S HOSPITAL.

A very neat and attractive uniform is worn by the nursing staff of St. Thomas's Hospital. The illustration represents a sister with her staff nurse and probationer. The sister, who occupies a seat in the centre of the little group, is attired in a dress of navy blue linen, simply relieved by a tiny white spot, and made quite plain. The skirt is full, and turned up round the bottom, with a substantial hem; and is in a small bow under the chin. The staff nurses' dress is made of narrow, blue striped galatea, which is always a favourite material. The skirt is quite plain, just clearing the ground, and is gathered into the bodice which buttons in front. The sleeves, like those of the sisters, are made wide enough to allow of the cuffs being worn underneath. The apron is plain linen of ample breadth, hemmed round the bottom, with a bib and straps attached, which cross at attached to the waistband by pleats or gathers. The bodice fits neatly to the figure and buttons in front. The sleeves, which are coat-shaped, are made wide enough at the wrist to admit of the cuffs being slipped on underneath. The collar is worn inside the band at the neck to match the cuffs. Over the dress comes a fine white linen apron hem-stitched all the way round, the square bib fastening to the bodice in front. The cap is made of cambric, which fits compactly to the head, and is drawn into shape by a runner at the back. Two rows of Valenciennes lace finish it off round the edge, forming a soft and becoming framework to the face. Strings, edged with lace to match, are brought from the sides, and tie the back and fasten at the waist. Simple spotted net forms the material for the cap. The shape is rather pointed in front, and is finished off round the edge with two rows of spotted net bordering, which is kept in position by a thread run along the reverse side. A pretty lilac and white striped print dress distinguishes the probationer from the staff nurse. It is made quite plain, with full skirt and tight-fitting bodice. The apron, like that worn by the staff nurse, is of plain linen, furnished with a bib and straps that cross over and fasten behind. A cap of similar shape and material completes the costume, making a charming *tout ensemble*. Our artist has hardly done justice to the caps which, as worn by the nursing staff of St. Thomas's, are both neat and becoming.

### Everybody's Opinion.

[Correspondence on all subjects is invited, but we cannot in any way be responsible for the opinions expressed by our correspondents. No communications can be entertained if the name and address of the correspondent is not given, or unless one side of the paper only be written on.]

### PRIVATE NURSING.

"A Nurse Matron" writes: I am much interested in a letter that appeared in The Hospital March 16th, entitled "Private Nursing," and I am very sorry that any of my fellow-workers are out of employment. At the same time, I fully endorse the sentiments of "L. W. R.," and think that the "modern trained nurse" makes a great mistake in refusing to take any part in what she styles menial work. One of the medical profession lately remarked to me, "What we need is a nurse who does not expect one or two maids in constant attendance on her." Nurses need not be household drudges, yet surely it would not affect their position if they manifested some interest in the domestic arrangements of a household; for example, by helping to keep the patient's room tidy, attending to the fire, and in various ways showing sympathy with the tired domestic, perhaps already overburdened with work, and who sometimes complains that "Nurse is always ringing that bell." I have had experience in private nursing, and have been in houses where it was absolutely necessary to superintend the cooking, otherwise the tired husband on his return home would find nothing fit to eat. I thought this part of my duty, and the wife, who was my patient, felt very grateful for this little assistance. If a nurse is asked to dine with the maids, where is her objection? Certainly one whose duty it is to minister to others should be able to accommodate herself to any society without feeling the worse for it. If a trained nurse be necessary in a sick chamber, she should take care not to become regarded as "a necessary evil" by creating work.

### ANOTHER PRIVATE NURSE.

"Nurse F. H." writes: I read with much interest the letter of "L. W. R" in your issue of March 16th. I have been a private nurse for some years, and can understand the feelings of others who object to undertake the entire charge of the invalid's room. Nurses who are called upon to clean the grate and other dirty work, cannot possibly keep their clothes and their hands nice enough to do the more delicate work of attending to the patient. Dusting and keeping the room in order is a different matter altogether, and one that few nurses object to. As to taking meals with the maids, the little time spent out of the sick room is not agreeably taken up by listening to differences which arise between mistress and maid. The possibility of the nurse's opinion on subjects of this kind being asked is not a pleasant one. So many people in the present day are constantly changing their servants that the latter are not always loyal to their employers. It is, therefore, quite comprehensible that nurses are not found willing to "take their meals with the maids," in spite of a good salary.

[Although "cleaning the grate" is spoken of as "dirty" work, we learn from several undeniably refined and accomplished private nurses that, with the modern appliances of polished bars, slow combustion grates, and other matters, a neat-handed woman can reduce "doing up the fire-place" to a small act of skilled labour. Thick gloves render even the softest of hands impervious to the dust of ashes or coal, and cannot be as harmful to the skin as the strong disinfectant solutions into which no trained nurse dare hesitate to immerse hands and arms whenever necessary. We agree with our numerous correspondents that no trained nurse should ever be asked to take her meals with the servants; and the employers who insist on such an arrangement must be content with the worthy old-time nurse, or the far less competent modern development, aptly designated "Helps Homeward."—Ed. *T. H.*]

### Our American Letter.

By Our Correspondent.

The nurses at Asbury Hospital, Minneapolis, recently proved their efficiency in dealing with emergencies after a most admirable fashion. A fire broke out one day in February, and, had it not been for the presence of mind which the nurses displayed, the consequences to the patients might have been fatal. However, all were safely removed from the scene of the fire, the typhoid cases being treated with due care, and handled with considerable skill and intelligence. Eventually the damage to the edifice was found to have been as much due to water as to fire.

A free bed at the Mary Hitchcock Hospital, Dartmouth College, has been endowed by the liberality of Miss Clare Hitchcock, who has given 5,000 dols. for the purpose.

New buildings have been designed which will replace the old Fort McHenry Hospital, and they will, of course, possess all the modern conveniences both in structure and fittings. The new hospital will be near, but not on, the exact site of the present one.

The most noteworthy of the new hospital buildings at present is that situated at Philadelphia, where Mr. John D. Lankeman has erected magnificent additions to the German Hospital, of which he is the president. It has fireproof ceilings, and handsome staircases, and contains seventy fine rooms. 250,000 dols. have been spent on this edifice, which, in the matter of appointments and appliances, will rank second to none in the U.S.A.

To those who could not attend the conference of superintendents, which was recently described in The Hospital, the papers read there and now appearing in the American journal, the *Trained Nurse*, have been most instructive. The article by Miss Lucy Drown, superintendent of nurses at Boston City Hospital, is, however, calculated to raise much discussion. The title is rather a long one, namely, "A Consideration of Methods for the Protection of Training Schools for Nurses, from Applicants who have been Discharged for Cause from other Schools."

No doubt it is a praiseworthy thing for a superintendent who, having had to get rid of an unsatisfactory nurse, strives to save other schools from the same applicant. But, surely there are many dangers in the method suggested by Miss Drown. Naturally such faults as theft, immorality, untruthfulness, may justify an extreme course, but there are many minor faults for which a superintendent may blame a nurse and decide not to retain her. Yet the dismissed candidate may be perfectly suited to another hospital if worked on different lines, and she may even prove a valued nurse amidst other surroundings. In such cases the distribution to other hospitals of condemnatory circulars would seem a refinement of cruelty. In what other branch of women's work would such a procedure be tolerated? And for the pursuit of the gentle art of nursing to be environed by precautions savouring rather of police espionage rather than of the "Golden Rule," seems pitiable. Whilst no pupil nurse who has been discharged from one school should be admitted to another till a satisfactory explanation of the cause for her departure has been stated, yet such an inquiry could surely be prosecuted even on this great continent in a more merciful and yet equally satisfactory fashion.

In Mrs. Hunter Robb's paper she applies the observations as to her own experience in the training of nurses at the Johns Hopkins Hospital to a scheme for the shortening of nurses' hours and the lengthening of their training.

[As soon as space permits we propose to publish the papers read at the Conference, of which copies have been placed in our hands.—Ed. *T. H.*]

## Figures and Tables

**Fig. 19 f1:**
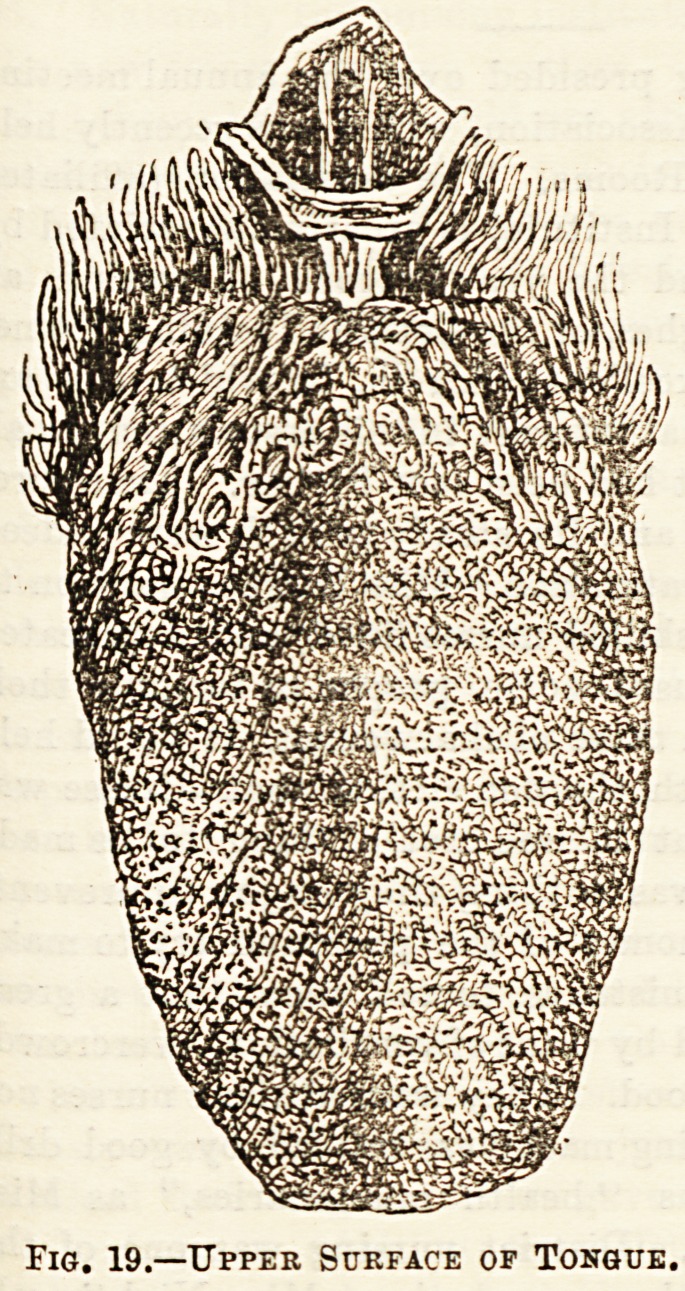


**Fig. 20 f2:**
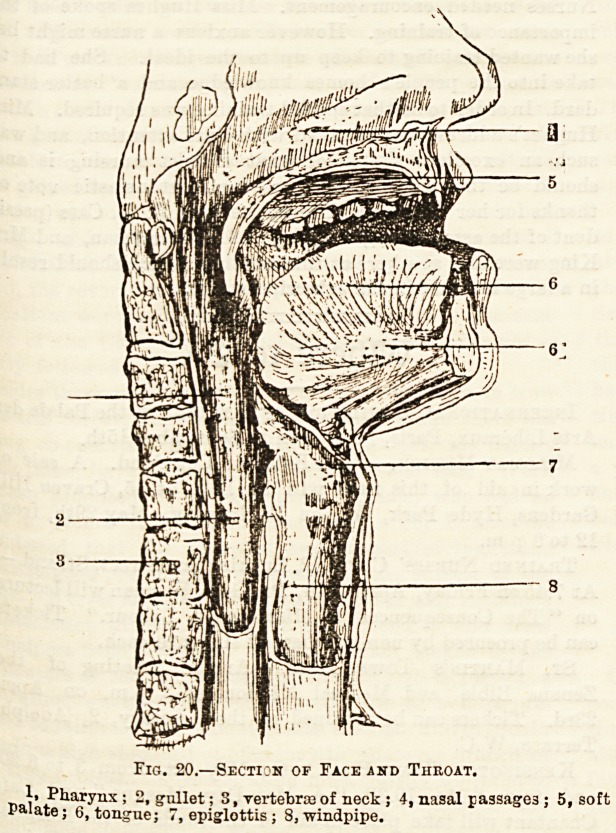


**Fig. 21 f3:**
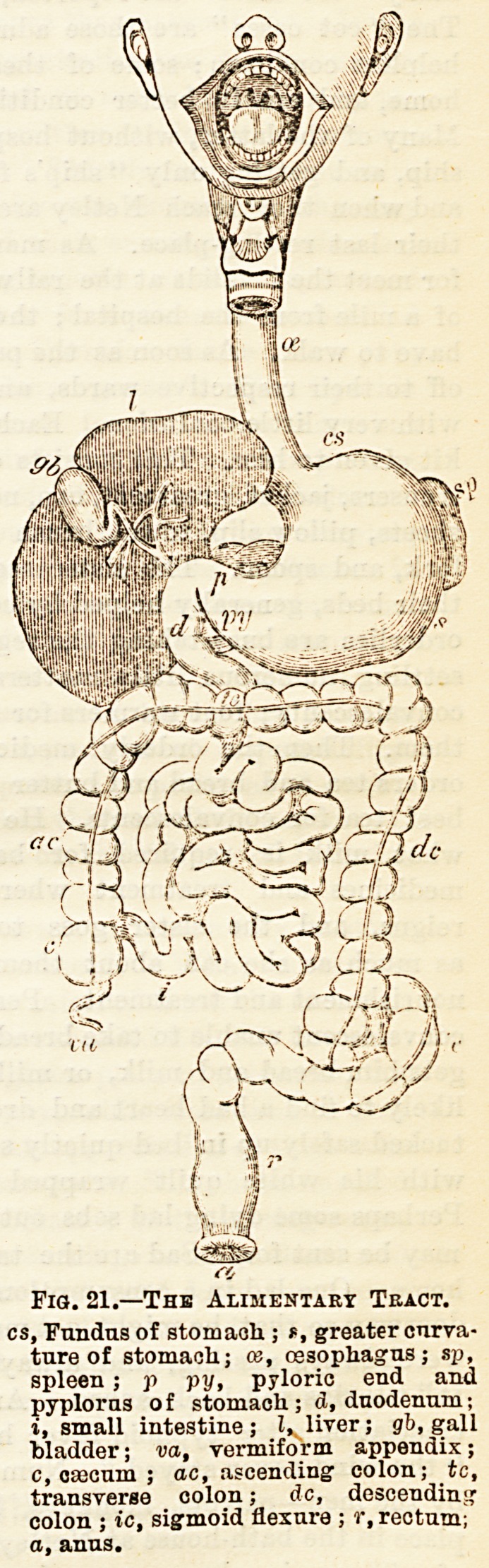


**Figure f4:**